# Venlafaxine-Induced Orthostatic Hypotension in a Geriatric Patient

**DOI:** 10.1155/2013/761567

**Published:** 2013-07-31

**Authors:** Vidyashree Chikkaramanjegowda, Jose de Leon

**Affiliations:** ^1^Department of Psychiatry, College of Medicine, University of Kentucky, Lexington, KY 40509, USA; ^2^University of Kentucky Mental Health Research Center, Eastern State Hospital, Lexington, KY 40508, USA

## Abstract

Venlafaxine is not usually associated with risk of orthostatic hypotension. A 65-year-old US Caucasian female taking 225 mg/day of venlafaxine extended-release developed symptomatic orthostatic hypotension. The systolic and diastolic blood pressure dropped by 25 and 18 mm Hg, respectively, from supine position to standing position within 3 minutes. The patient was otherwise healthy and the orthostatic hypotension resolved with venlafaxine discontinuation. This was a probable venlafaxine adverse drug reaction according to the Naranjo scale. This case contributes to the scarce literature that indicates that clinicians need to be aware that occasionally venlafaxine can induce clinically significant orthostatic hypotension, particularly in geriatric patients. Our patient did not have orthostatic hypotension when she was taking venlafaxine at 60 years of age in higher venlafaxine doses (300 mg/day) but developed this adverse drug reaction when venlafaxine was restarted at the geriatric age. This case indicates that a history of prior tolerance to venlafaxine does not guarantee tolerance after 65 years of age. If a clinician decides to use venlafaxine in geriatric patients, the clinician should warn the patient about the risk of orthostatic hypotension and consider very slow titration and low doses.

## 1. Introduction

The Consensus Committee of the American Autonomic Society and the American Academy of Neurology [1] defined orthostatic hypotension as “a reduction of systolic blood pressure (BP) of at least 20 mm Hg or diastolic BP of at least 10 mm Hg within 3 minutes of standing.” This consensus definition has recently been updated with minor changes [2].

 Orthostatic hypotension can be an adverse drug reaction (ADR) to several antidepressants and antipsychotics. These drugs can cause orthostatic hypotension during the first few weeks of treatment but usually tolerance develops. Thus, the risk for hypotension is one of the reasons that slow titration is recommended for some of these drugs. Orthostatic hypotension is usually explained by the alpha-_1_ antagonistic properties of some antidepressants and antipsychotics [3]. Among antidepressants, several of the tricyclic antidepressants (TCAs) are potent alpha-_1_ antagonists, but venlafaxine is usually considered to be lacking in antagonist properties at these receptors [3]. Articles focused on venlafaxine cardiovascular ADRs describe venlafaxine as being prone to cause hypertension, but it is not considered to cause orthostatic hypotension [4, 5]. The blockade of the noradrenergic reuptake transporter explains why venlafaxine can frequently cause hypertension [5].

 We described one case of venlafaxine-induced orthostatic hypotension that was carefully assessed using the Naranjo scale [6], one of the most frequently used ADR scales. This case contributes to the scarce literature that indicates that clinicians need to be aware that occasionally venlafaxine can induce clinically relevant orthostatic hypotension, particularly in geriatric patients. Moreover, our patient did not have orthostatic hypotension with prior venlafaxine treatment but developed this ADR when she reached geriatric age.

## 2. Case Presentation

 A 65-year-old US Caucasian female had a history of* DSM-IV-TR* major depressive disorder and anxiety disorder NOS. She had no significant medical problems. For three years (from 57 to 60 years of age), she was treated with 300 mg/day of venlafaxine extended-release (150 mg twice a day) and 10 mg of zolpidem at night. She had no ADRs, but, due to a change in the coverage of her medical insurance, she was switched from venlafaxine to citalopram (20 mg/day). After 6 months, she asked her psychiatrist to switch her antidepressant due to uncontrolled anxiety. For the next 2 years (from 60 to 62 years of age), she tried duloxetine (60 mg/day), but, after observing a worsening of tics, she was switched back to citalopram (20 mg/day) and she was stable for 3 years. At age 65, after worsening of her tics, the patient requested her psychiatrist (VC) to switch her to venlafaxine extended-release.

 The psychiatrist started venlafaxine extended-release 75 mg/day and recommended gradual titration to the target dose of 300 mg/day (150 mg administered twice a day) over 5-week duration. Citalopram was tapered off in 7 days. At 5-week followup, when the patient had been on venlafaxine for 4 weeks, including a dose of 225 mg/day for 14 days, the patient complained of “lightheadedness” and “blacking out,” particularly when rising from supine to standing position. This worsened with the increased dose of venlafaxine. The patient had no other medical problems; her only other medication was zolpidem at the same dose (10 mg at night) for the last 2 years. The psychiatrist established that the patient met criteria for orthostatic hypotension [1, 2] since the systolic and diastolic BP dropped by 25 and 18 mm Hg, respectively, from supine position to standing position within 3 minutes. The systolic BP dropped from 135 to 110 mm Hg while the diastolic dropped from 94 to 76 mm Hg. She was tapered off venlafaxine in 7 days and restarted on citalopram. One week later (3 days after the complete discontinuation of venlafaxine), the patient reported a complete resolution of the symptoms. In a follow-up visit (3 weeks after the complete resolution of symptoms), she did not have any significant change in her orthostatic blood pressure; her systolic BP dropped by 8 mm Hg (from 118 to 110 mm Hg) while the diastolic dropped by 7 mm Hg (from 69 to 62 mm Hg) from supine position to standing position within 3 minutes. The BP drop no longer met the criteria for orthostatic hypotension.

 The patient's psychiatrist (VC) scored the probability that this ADR was explained by venlafaxine treatment using the Naranjo scale [6]. She obtained a score of 8 (range up to 9) that indicated that the ADR was probably associated with venlafaxine treatment.

## 3. Discussion

 The Naranjo scale [6] indicated that the ADR was probably associated with venlafaxine treatment. A PubMed search using the terms “venlafaxine and orthostatic hypotension” provided a case report by Duggal et al. [7] and a geriatric study by Johnson et al. [8]. Duggal et al. described a clinically relevant orthostatic hypotension case in a 46-year-old Indian male with two episodes of falls on 225 mg/day of venlafaxine extended-release [7]. They did not provide a score using the Naranjo scale. With the available published information, one of us (VC) obtained a score of 7 indicating a probable venlafaxine ADR. Johnson et al. [8] described an open-label 12-week study on 72 geriatric patients taking venlafaxine. They measured orthostatic changes in 47 of their patients weekly. There were 38 patients who had no orthostatic changes at baseline before starting venlafaxine; 50% of them had orthostatic changes at least twice during the 12 weeks. More importantly, of the 30 patients who did not endorse dizziness at baseline, 8 developed mild dizziness during venlafaxine treatment. Although other cardiovascular ADRs were associated with venlafaxine discontinuation, no patient discontinued venlafaxine due to orthostatic changes and/or dizziness. It was recommended that patients with orthostatic changes drink more fluids and go from supine to standing position slowly. Thus, this recommendation appeared sufficient to avoid symptomatic orthostatic changes.

Duggal et al. [7] provided a reference for a relevant Australian venlafaxine study [9] not included in PubMed. Alderman and Wiese [9] described a retrospective review of 13 of 18 (72%) mainly geriatric patients who unexpectedly had orthostatic hypotension during titration. Contributing factors included (1) history of hypertension, or ischaemic heart disease, and (2) intake of antihypertensives, or nitrate vasodilators.

 As antidepressants typically cause orthostatic hypotension by blocking alpha-_1_ adrenergic receptors [3], it is not unreasonable to suspect that this case may be also explained by venlafaxine blockade of these receptors. *In vitro* studies usually state that venlafaxine has no affinity for alpha-_1_ adrenergic receptors [3], but the affinity of venlafaxine metabolites for these receptors has never been studied, to the best of our knowledge. Some cases of lethal venlafaxine overdoses were associated with refractory hypotension [10], indicating that something associated with venlafaxine treatment may cause, in special circumstances, clinically relevant alpha-_1_ adrenergic blockade.

 Our case is interesting in the sense that our patient did not develop orthostatic hypotension when she previously took venlafaxine (from 57 to 60 years of age), but she developed it when she reached 65 years of age. Geriatric age is frequently associated with clinically relevant changes in pharmacokinetics [11] and pharmacodynamics [12]. The decrease in renal drug clearance may be the most important factor related to venlafaxine, since geriatric patients tend to have 50% higher plasma venlafaxine concentrations [13]. Geriatric age is associated with pharmacodynamic changes that are not well understood [12]. According to Trifiró and Spina [12], one of the most evident pharmacodynamic geriatric changes is the selective dysfunction of the homeostatic systems that control the alpha-_1_ adrenergic receptor system, which mediates acute changes in vasoconstriction. Thus, it can be postulated that during the last 5 years, our patient progressively developed a decrease in renal clearance and dysfunction of the alpha_1_-adrenergic receptor vascular system, which explained her orthostatic changes on venlafaxine at 65 years of age, despite no symptoms when she was younger.

 In spite of the fact that venlafaxine is not expected to cause orthostatic hypotension, in some patients orthostatic hypotension may not only be clinically relevant but can be associated with substantial risks, particularly of falls. Our case is unique since the patient did not have orthostatic hypotension with prior venlafaxine treatment. However, when the patient was restarted on venlafaxine at 65 years of age, she was no longer able to tolerate lower venlafaxine doses than those she had taken 5 years earlier. Our case contributes to the literature that indicates the substantial risk of orthostatic hypotension in geriatric patients treated with venlafaxine. If a clinician decides to use venlafaxine in geriatric patients, the clinician should warn the patient about the risk of orthostatic hypotension and consider very slow titration and low doses. A history of prior tolerance to venlafaxine does not imply tolerance after 65 years of age.
